# The metabolic addiction of cancer stem cells

**DOI:** 10.3389/fonc.2022.955892

**Published:** 2022-07-25

**Authors:** Om Saswat Sahoo, Karthikeyan Pethusamy, Tryambak P. Srivastava, Joyeeta Talukdar, Mohammed S. Alqahtani, Mohamed Abbas, Ruby Dhar, Subhradip Karmakar

**Affiliations:** ^1^ Department of Biotechnology, National Institute of technology, Durgapur, India; ^2^ Department of Biochemistry, All India Institute of Medical Sciences, New Delhi, India; ^3^ Radiological Sciences Department, College of Applied Medical Sciences, King Khalid University, Abha, Saudi Arabia; ^4^ BioImaging Unit, Space Research Centre, Michael Atiyah Building, University of Leicester, Leicester, United Kingdom; ^5^ Electrical Engineering Department, College of Engineering, King Khalid University, Abha, Saudi Arabia; ^6^ Computers and communications Department, College of Engineering, Delta University for Science and Technology, Gamasa, Egypt

**Keywords:** cancer stem cells, metabolism, epigenetics, cross talks, oncometabolites

## Abstract

Cancer stem cells (CSC) are the minor population of cancer originating cells that have the capacity of self-renewal, differentiation, and tumorigenicity (when transplanted into an immunocompromised animal). These low-copy number cell populations are believed to be resistant to conventional chemo and radiotherapy. It was reported that metabolic adaptation of these elusive cell populations is to a large extent responsible for their survival and distant metastasis. Warburg effect is a hallmark of most cancer in which the cancer cells prefer to metabolize glucose anaerobically, even under normoxic conditions. Warburg’s aerobic glycolysis produces ATP efficiently promoting cell proliferation by reprogramming metabolism to increase glucose uptake and stimulating lactate production. This metabolic adaptation also seems to contribute to chemoresistance and immune evasion, a prerequisite for cancer cell survival and proliferation. Though we know a lot about metabolic fine-tuning in cancer, what is still in shadow is the identity of upstream regulators that orchestrates this process. Epigenetic modification of key metabolic enzymes seems to play a decisive role in this. By altering the metabolic flux, cancer cells polarize the biochemical reactions to selectively generate “onco-metabolites” that provide an added advantage for cell proliferation and survival. In this review, we explored the metabolic-epigenetic circuity in relation to cancer growth and proliferation and establish the fact how cancer cells may be addicted to specific metabolic pathways to meet their needs. Interestingly, even the immune system is re-calibrated to adapt to this altered scenario. Knowing the details is crucial for selective targeting of cancer stem cells by choking the rate-limiting stems and crucial branch points, preventing the formation of onco-metabolites.

## Introduction

Cancer stem cells (CSCs), also known as tumor-initiating cells, constitute a rare subset of cells in cancerous tumors, characterized by an enhanced capacity for self-renewal, multipotency, tumor initiation, and tolerating foreign niches required for the growth of the tumor bulk. Located at the apex of the pyramid of tumor cells, they foster the very nature of malignancy and thus play a vital role in regenerating the heterogeneous cell population, unrestrained proliferation, metastatic dissemination, and sustaining therapy.

CSCs were first identified in AML, using cell surface markers, followed by other solid malignancies such as brain, breast, colon, pancreas, etc. Despite their heterogeneity amidst different tumor bulks, CSCs of almost all the subsets possess identical genetic backgrounds *via* the acquisition of stemness genes, expression of markers like CD44, CD133, ALDH, activation of specific signaling pathways, etc. As a result, the study of CSCs has not been restricted to just cell surface markers; instead, other complementary methods have been envisaged to measure the functional activation of CSCs on a molecular level. Accumulating shreds of evidence have also suggested striking parallels between metabolic phenotypes and CSCs in particular. Metabolic vulnerability is believed to be the hallmarks of these CSCs, thus providing another broad field of research. Yet another novel field of CSC interactions is how the epigenetic system harnesses tumorigenesis and stemness of the CSCs and cancer cells. However, another element of the tumor microenvironment is the immune system in tumorigenesis, which plays a supportive and inhibitive role in the development of CSCs an d cancer. Though these three domains of CSCs stay as integral elements of the tumor microenvironment, they participate quite inclusively in tumorigenesis. Metabolic addictions, epigenetic modifications and the pro/anti tumorigenic properties of the immune system play hand in hand to develop the framework of CSCs and the cancer cells, thus establishing cancer to be one of the leading causes of death globally.

Analogous to a hive’s queen bee, that the queen bees produce and nurture worker bees, killing the queen bee has always led to the hive’s demise. Similarly, destroying the CSCs has always been the appeal to eradicate tumor bulks. Though having an aberrant clinical behavior, several studies have helped determine the prognostic significance of CSCs and thus have suggested profound implications for cancer treatments. Combinational therapies of intra/inter phenotypic features of CSCs have been the demand for treating CSCs, because of its relapsing nature. In this perspective, the emerging fields of the CSC domains, viz CSC metabolism, CSC epigenetics, and CSC immunology, have been elucidated. Although domain-wise reviews are available in the literature, no collective review has been made to put forward into a single picture. In the present study, an effort has been made to congregate the epitranscriptional and immunogenic orchestration of genetic and metabolic programming and how they further impede the CSC microenvironment. The significance of biomarkers to detect CSC and cancer bulk populations has also been discussed. Further, the review will discuss the prospects of targeting all the above phenotypes for CSC therapy.

## Cancer stem cell metabolism

Since the discovery of CSCs, the role of metabolism in such cells has profoundly been studied. It has been conclusively demonstrated that the origins of these CSCs are substantial because of the upsurge of mutational events occurring in non-tumor cells, further characterized by elevated metabolic activities and plasticity. Characterized by high proliferation rates, their energy demand usually leads to increased biosynthesis of macromolecules and tight regulation of the cellular redox status (1). Thereby, they can be designated as metabolic omnivores, as they can sustain on a wide range of substrates to produce ATP molecules. Moreover, it has been observed that these CSCs have a noticeable ability to adapt to changes according to their physiological environment by selecting the most efficient substrate.

CSCs follow a different metabolic phenotype; however, their characterization is still contentious (2). With discrepancies in the biological function of normal differentiated tumor cells and CSCs, it has been observed that CSCs mimic the metabolic phenotype of the stem cell hierarchy. It was expected that CSC metabolism would conversely orient towards mitochondrial OXPHOS, unlike the widely accepted aerobic glycolysis (Warburg effect) (3). However, only a few studies support the fact that the primary metabolic phenotype of CSCs is mitochondrial OXPHOS. Results based on samples of glioma (4), PDAC (5) and leukemia stem cells (6) show a positive response; still, data obtained from breast CSC samples show discouraging responses in this regard (7).

Further, another misleading interpretation comes from the fact that cell cultures are based on non-physiological high glucose concentrations; thereby, there are possibilities that CSCs must be shifting their primary metabolic phenotype to adapt to the environmental changes. Factors such as biochemical reactions, paracrine cross-communications, and local ecological factors actively stimulate self-renewal pathways for maintaining a CSC niche (8). Though evidence for glucose-based and mitochondrial oxidation has been broadly accepted, it has also been observed that lipids and amino acids, like lysine and glutamine, may also act as alternative metabolic fuels for CSCs (9). The following section describes the above in detail.

## Metabolic profile of CSCs

### Glycolytic pathway

CSCs usually exhibit an enhanced glucose metabolism pathway, with higher glucose uptake, lactate production, expression of glycolytic enzymes, and high ATP content. Several studies show that CSCs tend to follow a glycolytic pathway to meet their glucose demands. In certain tumors, it has also been observed that deprivation in glucose intake induces *in vitro* depletion of CSCs. The increase in the expression of proteins and other factors associated with glucose metabolism confers a longer lifespan for CSCs (10). This was further discussed by Cluntun et al., that CSCs rely on glutamine metabolism, too as glutamines are carbon and amino-nitrogen sources (11).

CSCs from CD133+ hepatic, breast, and nasopharyngeal carcinoma shows a preferential inclination towards aerobic glycolysis (12). Methylation of Fructose Biphosphate-1, FBP1 and other gluconeogenesis enzymes has been seen to help glycolysis retain stemness features in an aggressive form of breast cancer, BLBC (13). However, overexpression of FBP-1 and an increase of ROS have shown commendable results in the reduction of CD44^high^/CD24^low^/EpCAM^+^ in BLBC, thereby reducing their tumor-forming capability *in vivo* (13). This phenomenon was later amended by Peng et al. that BLBCs show profound levels of PDK 1, which is known to inhibit mitochondrial OXPHOS (14). A depletion of PDK1 drastically decreases the presence of ALDH-1 positive BLBCs, thereby decreasing tumor spheroid forming ability. A similar response was also noted when snail-mediated lowering of cytochrome C oxidase and FBP1 expression inhibited OXPHOS and activated glycolysis (15). Snail also plays a vital role in regulating glucose flux and PPP by inhibiting phosphofructokinase platelets (PFKPs), under conditions with nutrient deficiency (16). It has also been profoundly noticed that CSCs exhibit an upregulation in glycolytic enzymes and gene expressions like GLUT1, HK-1, HK-2, PDK-1, c-Myc, CD-44, and PGAM-1 along with downregulation of gluconeogenic enzymes like G6PC and PEPCK (17, 18). Hsp90, in coordination with other epigenetic factors like PKM2, also has been seen to mediate the metabolic signature of CSCs and promote glycolysis (19). PKM2 following Oct4 and β-catenin has been seen to express glycolytic gene expression, hence promoting stemness quality in cells (20–22). Many hypotheses have also confirmed the role of hypoxia as one of the characteristic features of the CSC niche. Glucose has a lower diffusion rate than oxygen; however, the solubility of glucose is higher than that of oxygen, which also delineates the credibility of a glucose-based metabolism in CSCs. Thereby, in a hypoxic niche, the cells compensate for their low oxygen conditions by inducing HIFs, which mediate the expression of a considerable number of genes involved in this regard. HIF-1α and the mTOR/Akt/beta-catenin stem cell regulatory pathway stimulate the transcription of the essential GLUTs and almost all the glycolytic enzymes, namely, LDH, GLUT, HK2, MCTs thereby endorsing that hypoxic conditions favor a glycolytic based metabolism (23–25).

In sum, the above observations help us postulate that activation of the glycolytic phenotype favors stemness in CSCs.

### OXPHOS and mitochondrial biogenesis

Despite the above-reported studies, an overwhelming amount of publications signify that CSCs use mitochondrial biogenesis and OXPHOS predominantly. In fact, Sato et al., in their experiment, demonstrated the reprogrammed metabolome analysis of the CSCs *via* the TCA cycle (26). Compared to non-CSCs, it was determined that activation of TCA cycles could be a characteristic feature of CSCs. A similar trend was observed in small lung cancer cells when CSCs were compared based on dependence upon OXPHOS and mitochondrial function (27). Convincing results were also reported concerning CD133+ glioblastoma (28), ROS^low^ quiescent leukemia stem cells (6), ovarian (29), and PDAC (30) to privilege OXPHOS as their metabolic phenotype. Furthermore, Janiszewska et al. showed that glioma-spheres were outlined by oncofetal insulin-like growth factor 2 mRNA-binding protein 2 (IMP2), a pivotal OXPHOS regulating factor (28). A study performed on CD133^+^ CD49f^+^ hepatocellular carcinoma cells showed that NANOG’s pluripotency factor contributes towards reprogramming mitochondrial metabolism to meet cellular demands (31). NANOG ChIP-seq analysis and metabolomic profiling determined that OXPHOS and FAO are NANOG-mediated oncogenic pathways. NANOG silencing leads to increased OXPHOS activity and downregulation of both transcripts and protein levels of FAO-associated genes.

Yet another study found out that transcription co-activator PGC-1α is a vital factor in stimulating mitochondrial biogenesis and OXPHOS, enhancing the cancer cells’ metastatic, migratory and invasive capability (32, 33). However, it has also been observed that inhibition of PGC-1α leads to a reduction of stemness properties in breast CSCs (32–35). Interestingly, overexpression of MYC genes curbed stemness properties *via* negatively controlling the expression of PGC-1α. It has been observed in breast tumor cells that this c-Myc oncoprotein has been shown to promote mitochondrial glutaminolysis as a part of its reprogramming of cellular metabolism. Being a global transcriptional regulator, MYC significantly induces changes in mitochondrial morphology and dynamics to promote the biosynthesis of metabolic precursors and cell development. MYC establishes a stereotypic gene expression in human tumors and has an inverse relationship with YAP/TAZ activity to drive clonogenic growth (36). Apart from these epigenetic factors, signaling pathways like Notch, PI3K/Akt, PTEN, NF-ĸB, Wnt/β-catenin, KRAS, and HIFs also influence CSC metabolism (37).

Studies also show that the metabolic profile of epithelial ovarian CSCs is characterized by high glucose intake and elevated PPP and OXPHOS. Elevated OXPHOS and reduced glycolysis activity were also deliberately expressed by high telomerase activity (hTERT-high) CSCs, isolated from lung and ovarian cancer models (38). The CD44^+^CD117^+^ cells of the ovary give rise to a substantial amount of mitochondrial reactive oxygen species (ROS) and die when the mitochondrial respiratory chain is blocked (29). However, research shows that CSCs show a significantly lower amount of ROS, reflecting that CSCs have enhanced ROS defenses, contributing to an increased expression of free radical scavenging system compared with their non-tumorigenic cells counterparts (39).

Several other mitochondrial carriers like SLC25A10 and NT5M were seen to be upregulated in varied cancer cell lines. Further analysis has shown that this SLC25A10 upregulation dysregulates a diverse amount of processes such as gluconeogenesis, urea cycle, FAO etc. However, overexpression of NT5M has been seen in maintaining dTTP levels of mitochondria, hence maintaining mitochondrial DNA synthesis and preserving its DNA copy number. Other genes associated with dTTP syntheses like RRM2, TYMS, and TK1 have also been seen to be overexpressed (as high as 20-folds) in cancer cell lines *in vivo*. This elevated dTTP pool plays a vital role in providing genetic stability, counteracting the imbalances occurring in the mitochondria thus protecting the mitochondrial genome (40).

Apart from generating ATP molecules for cancer cells, mitochondria communicate with the cytosol, release cytochrome C to commence cell death, and release ROS and other metabolites (41–43). They also appear to be responsible for regulating the stemness of CSCs irrespective of the metabolic phenotype of the CSC (27, 30, 39). Based on the above observations, it can be deduced that mitochondrial health plays a pivotal role in the maintenance of CSCs.

### Alternative metabolic phenotypes of CSCs

CSCs also happen to rely on metabolic phenotypes, other than glycolysis and OXPHOS to sustain self-renewal, maintain stemness features and avoid anoikis. As mentioned above, CSCs from hepatocellular carcinoma cells rely on FAO as one of the metabolic phenotypes (31). Pasto et al. also stated an overexpression of genes associated with PPP and FAO in ovarian CD44^+^CD117^+^ cells (29). Promoters of FAO genes like Acads (Acyl CoA dehydrogenase short chains) and ECHS1 (enoyl-CoA hydratase short chain 1) have been seen to bind with NANOG and trigger lipid biosynthesis to maintain stemness in CSCs (44). Upregulation of CD36 also plays a vital role in fatty acid import, thus promoting fatty acid synthesis. Recent advances in proteomics have indicated that fatty acid metabolisms play an essential role in redox homeostasis and determining the metabolic fate of CSCs. Mitochondrial FAO also plays a pivotal role in the epigenetic regulation of gene transcriptional factors. Key enzymes of lipogenesis like SREBP1 (45–47), FASN (48), CPT1 (49) have been overexpressed in many forms of human cancers. Upon activation of EGFR signaling, the interaction between SREBP1 and PKM2 potentiates SREBP genes to turn active, triggering lipid biosynthesis (44).

In addition, metabolic rewiring between PPP and glycolysis has also been noticed in glioblastoma CSCs. PPP enzymes were overexpressed in acute oxygenation regions and under-expressed in the hypoxia-mediated areas, unlike glycolysis-based enzymes, where the reverse occurs (50). Intriguingly, glutamine has been identified as an EMT-associated metabolite, demonstrating its role in CSCs from several tumors (26, 51). Recent studies have revealed that the c-Myc gene and Dlx2/GLS1/Glutamine axis enhances glutaminase expression (the enzyme responsible for catalyzing glutamine to glutamate), thereby suggesting the oncogenic signature of glutamine metabolism (52, 53). The glutamine uptake in CSCs has also been associated with upregulation of alanine, serine, cysteine-referring transporter 2 (ASCT2). Glutamate oxaloacetate transaminases (GOT1 and GOT2), enzymes that are responsible for converting glutamate-derived aspartate to oxaloacetate, also show an elevated expression in CSCs (54).

### Epigenetics of CSCs: Does it rewire the CSC metabolism?

**Figure 1:**
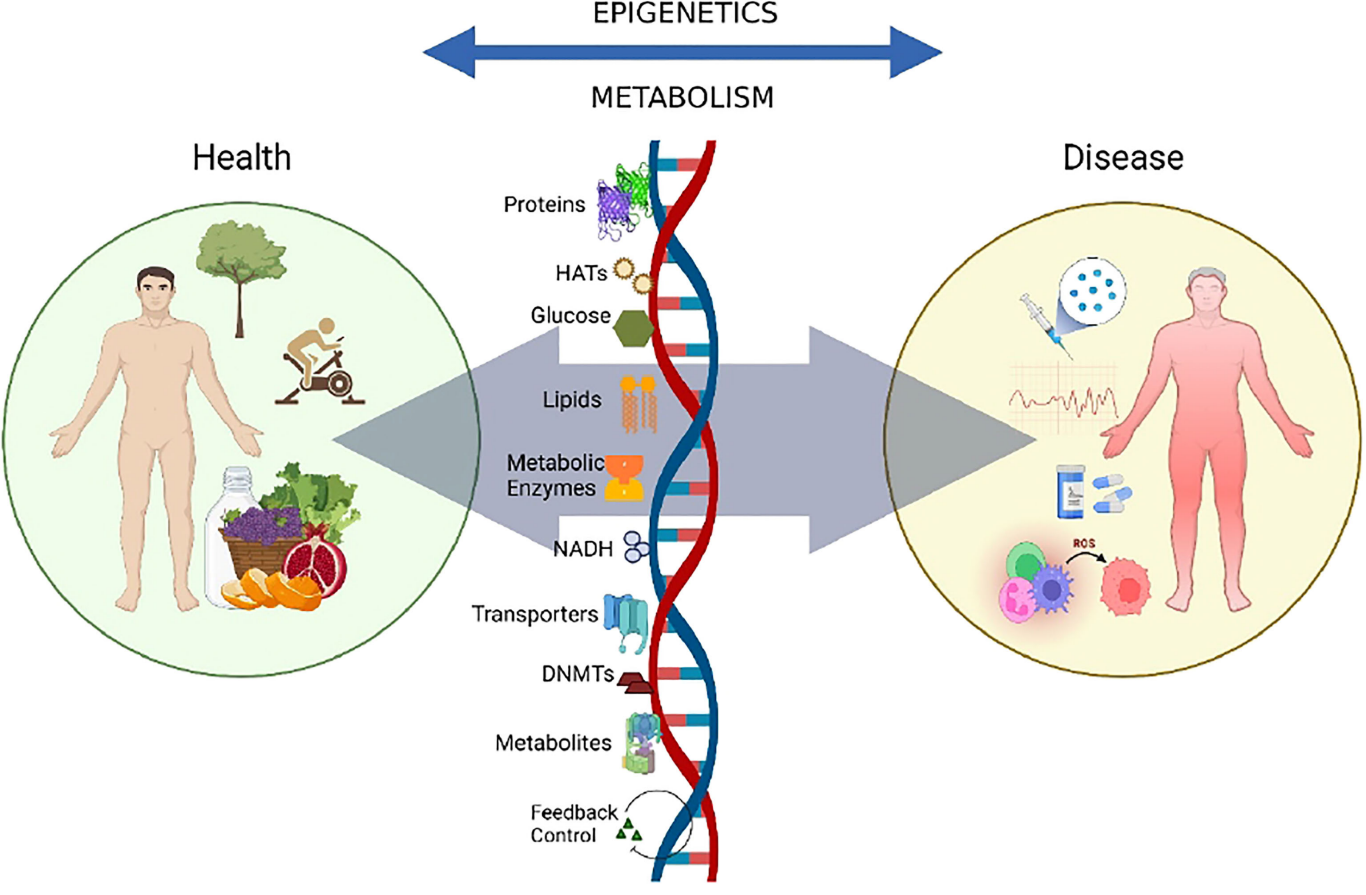
Crosstalk between epigenetics and metabolism.The crosstalk between epigenetics and metabolism is implicated in a variety of contexts related to disease progression. The availability of modifiers like metabolites, co-factors, chromatin-modifying enzymes, and other environmental factors like nutrition, exercise, and the gut microbiome, modulate the dynamics of the genome, thus contributing towards metabolic and epigenetic control and disease progression.

Epigenetic regulation governs a cell’s fate, followed by chromatin remodeling, leading to a normal somatic cell becoming its stem cell counterpart. A similar change in the epigenetic landscape in tumor-initiating cells can give rise to potential CSCs. Epigenetic modulation usually occurs *via* three mechanisms, DNA methylation, histone modification, and chromatin remodeling; however, several other methods have been identified in the recently (**Figure 1**). However, it has also been noticed that aberrations in these epigenetic/genetic factors and several cellular signaling pathways, demonstrating that these pathways also choreograph the metabostemness (as coined by Menendez), thus manipulating the metabolic programming of CSCs intrinsically. As further explained by Waddington’s buffering and canalization theory, metabostemness is driven by basically 2 methods; the first including the modification and methylation of DNA and histones, accounting to the reprogramming of metabolism and the second, comprising of the oncometabolite which decipher their presence in the form of chromatin modeling. This reprogramming also encompasses the impairment of the hallmark tumour microenvironment features thus remodeling both the epigenetic landscape and the dynamic metabotypes of CSCs (55). This epigenetic mediated switching of metabolic phenotypes also intervenes with the energy blockades enunciated during CSC treatment protocols, thereby promoting the flexible metabolic bonafide of CSCs and hence expanding their resilience of stemness. The further section describes the various reports of how epigenetic codes influenced the metabolic fates of CSCs and potentiated a positive feedback loop for signaling pathways in CSCs.

### Role of signaling pathways in CSCs influencing metabolism

**Figure 2:**
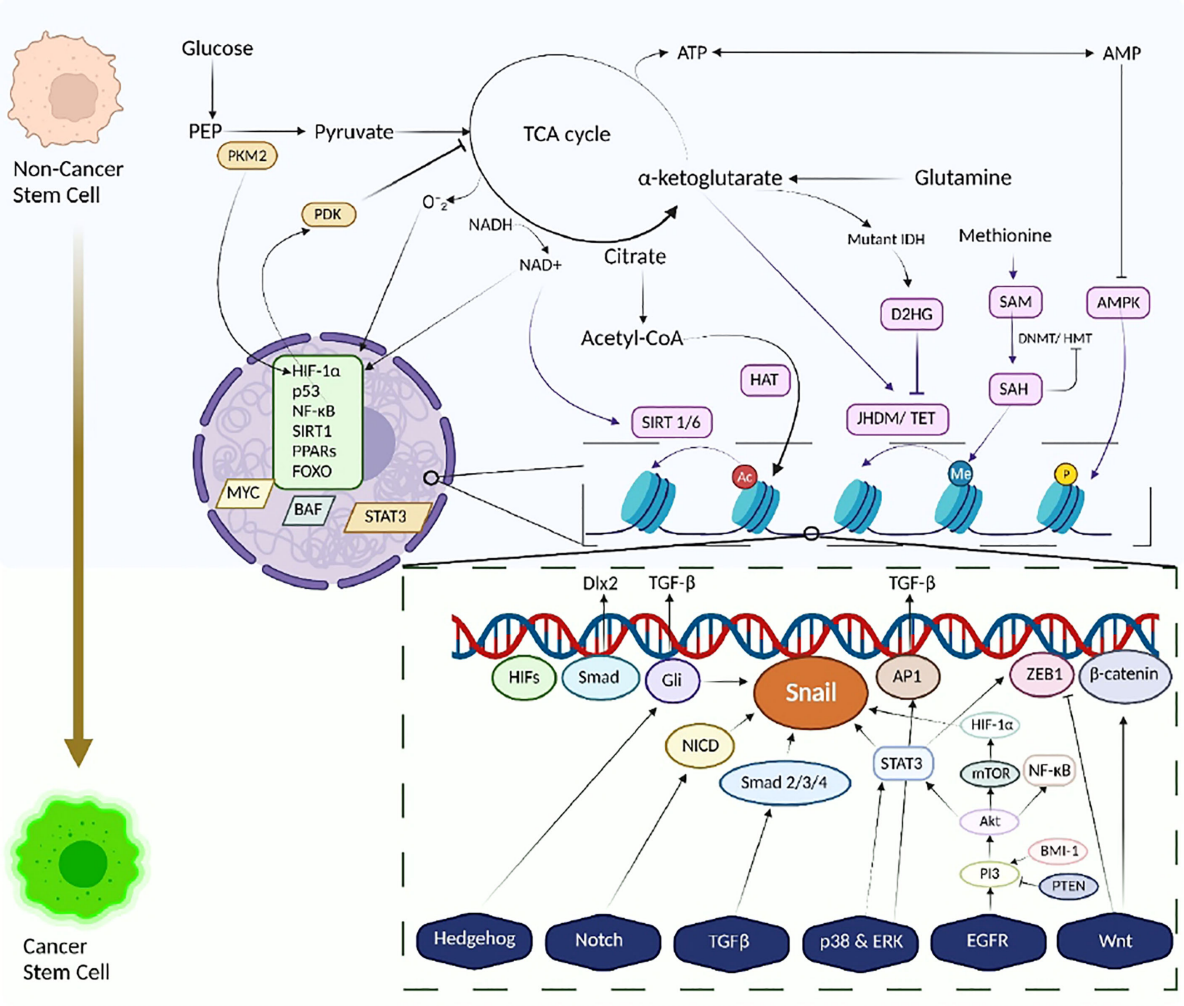
The metabolism-epigenetics axis of Cancer Stem Cells. Transformation of Stem cell to Cancer Stem cells along with metabolic reprogramming and Tumour metabolome modulates and links energy-generating biochemical reactions with several epigenetic pathways, thus integrating metabolism and a variety of signaling pathways with epigenetic modifications, histone changes. Signaling pathways involving HIF-1α and p53 dysregulate glucose and glutamine metabolism, contributing to enhanced production of acetyl Co-A and α-ketoglutarate. This enhanced production influences HAT activity, hence increasing the acetylation profile of cancer cells. A similar response is also observed in NADH/ NAD+ activity wherein the activity of Sirtuins changes. Other metabolites like D2HG and SAM, also play a vital role in epigenetic modifications, eliciting and inhibiting HMT/ DNMT activities. Energetic stress is also observed due to activation of AMPK leading to histone phosphorylation. The dysregulation of the metabolic homeostasis, in accordance with aberrant signaling pathways or mutations and epigenetic modifications, reprograms the stemness and pluripotency of the normal/ cancer cells towards cancer stem cells. Cancer stem cells exploit these altered metabolic pathways for their benefit and survival.

A complex array of metabolic pathways generating different metabolites not only carried the flux of matter but also bioactive molecules that can influence other metabolic pathways or signaling cascades (**Figure 2**).

### Wnt signaling

The Wnt signaling cascade is a mysterious complex encompassing 19 Wnt ligands and more than 15 receptors orchestrating many developmental processes like regulating cell homeostasis and maintaining adult stem cells in their pluripotent state (56). It encompasses three signaling pathways, canonical (mediated through β-catenin-T cell-specific transcription factor), non-canonical (independent of a β-catenin-T cell-specific transcription factor), and non-canonical Wnt-calcium pathway (regulates intracellular calcium levels) (57). The canonical pathway activates when Wnt ligands bind to Frizzled receptors, a G-protein-coupled receptor family, and a low-density lipoprotein related protein (LRP 5 and 6) on a neighboring cell (58). After the Wnt signaling gets activated, the β-catenin drives cellular responses *via* the transactivation of target genes and LEF/TCF transcription factor (59).

Aberrant activation of the Wnt/β-catenin pathway has also been linked with DNA methylation through subsequent silencing of various Wnt inhibitors like WIF-1, AXIN2, SFRP-1 and DKK1 in breast and CRCs (60–62). Methylation of Wnt negative regulators like DKK3 and NKD1 has also been correlated with gastric cancers (63). Apart from methylations, dysregulation of the pathway is also mediated by several histone modifications. Recruitment of SIRT1, EZH2 and PCR2 and decreased acetylation of H3K16 and increased H3K27 trimethylation has been eminent with deregulation of several Wnt antagonists (64). Wang et al. demonstrated the relevance of lncRNAs as epigenetic modulators of the Wnt signaling pathway, when they found lncRNAs of lncTCF7 induced TCF7 expression by recruiting SWI/SNF chromatin remodeling in liver CSCs, thus promoting self-renewal and tumorigenesis in the liver (65).

Experimental evidence suggests that Wnt signaling is activated in several malignancies, including CRC, breast, leukemia, lymphoma, medulloblastoma, and hepatocellular (66). In addition, Wnt activation has been seen in non-melanoma cutaneous tumor cells of murine models and potentially in humans (67). It also exerts influence over the glycolysis by upregulating Myc, MCTs and PDK. Distal-less homeobox -2 (Dlx-2), a protein-coding gene, also has been seen to trigger the glycolytic switch stimulated by Wnt/TGF-β and represses mitochondrial biogenesis by upregulating expression of snail and inhibiting mitochondrial complex IV, i.e. COX. Glutaminase-1, a predominant molecule expressed during glutamine metabolism has been seen to be induced by Wnt/TGF-β in a Dlx-2 dependent pathway (68).

### Hedgehog signaling

The Hedgehog (Hh) signaling pathway is essential for developing embryos, tissue homeostasis, and EMT transition (69). The three HH homologs of the HH pathway – Sonic, Desert, and Indian mediate the Patched receptor’s (PTCH1) inhibitory effect on Smoothened (SMO), thereby allowing Gli transcription proteins to get isolated. When activated, Gli proteins (Gli-1 and Gli-2) are released to facilitate transcription of target genes, thereby mediating the HH signaling transduction. It has been observed that the HH pathway gets stimulated through up-regulation of Gli by several intracellular signals, which are conveyed by several other epigenetic factors and signaling pathways like KRAS-MAPK/RAF/MEK, mTOR, TGF-β, Snf5, and Phosphoinositide 3-kinase/Akt pathway (70–74). In addition, histone modifications and DNA methylations also play a major role in regulating the HH pathway. Enhanced expressions of the Shh ligands have been seen in breast and gastric cancers due to hypomethylation of the Shh promoter (75, 76). Similar activity with NF-ĸB led to the activation and transcription of Shh, resulting in the upregulation of the ligand, thus promoting self-renewal and invasiveness of varied cancers (77). The Gli proteins have also been associated with HDAC1 in virtue of a positive autoregulatory loop, thus dysregulating neural progenitor and tumor cells (78).

However, deregulations in this HH pathway implicate tumorigenesis and tumor growth. Several studies have suggested the role of HH signaling in various CSCs, including basal cell carcinoma (BCC), medulloblastoma, rhabdomyosarcoma, glioblastoma, colon cancer, and chronic myeloid leukemia (CMC) (79–81). In addition, HH signaling was also seen in glioma spheres of nude mice and humans (82). Concerning metabolism, the HH pathway has been associated with lncRNAs like breast cancer anti-estrogen resistance 4 (BCAR4), a downstream target of YAP. It enhances the expression of YAP-dependent glycolysis activators, viz. HK2 and PFKFB3, thus making YAP/BCAR4 signaling axis a potential target for breast cancer treatment (83). PKM2 also has been seen to be associated with transcriptional repressor TGF- β-induced factor homeobox 2 (TGIF2) to repress the transcription of E-cadherin by inducing HDAC3.

### Notch pathway

Like Wnt and HH pathway, Notch signaling occurs between neighboring cells *via* transmembrane protein ligands (Delta 1/3/4 and Jagged 1/2) (84, 85). The interaction initiates by binding the ligands, which cleaves the Notch intracellular- domain (NICD), by -secretase. NICD then translocates into the nucleus, interacts with recombination signal binding protein for immunoglobin kappa J region (RBPJ-ĸ) to finally activate transcriptional factors, like MYC and HES1 (86, 87). It is regarded as highly conserved, it helps regulate cell fate specification, stem cell differentiation, stem cell renewal and triggers multiple aspects of cancers. Aberrations in the Notch signaling pathway have been shown to regulate tumor progressions in leukemia, breast, colon (CRC), pancreas, glioblastoma, lungs, and multiple myeloma. Notch has also been seen to synergize with HIF-1α and induce metastasis, and also maintaining stemness characteristics. Neural and breast CSCs show overexpression of the notch genes, leading to increased formation of progenitor cells. On the contrary, several studies also support the role of Notch signaling as a tumor suppressor. They have been seen to impair epidermal differentiation and skin barrier integrity in skin cancers. In another study, Notch signaling has been shown to induce cell death by increasing p53 activity in cancers like HCC, cervical cancers, and Ewing’s sarcoma (88). Overexpression of Jagged2 was also associated with HDACs leading to subsequent activation of Notch signalling in multiple myeloma (89). Jin et al. also demonstrated the interaction of serine-threonine kinase receptor-associated protein (STRAP) with EZH2 and PRC2 complex, inhibiting histone methylation of H3K27 on HES1 and HES5 promoters, further leading to regulating stemness potential in CSCs (90).

In addition to modulating the epigenetic landscapes of CSCs, the role of Notch signaling has also been seen in regulating metabolites and metabolic enzymes both directly and indirectly. Notch signaling stimulated transcriptional factors like HIF-1α, MYC, p53, and many others show active participation in conferring advantages in virtue of metabolic pathways for survival and proliferation of both cancer bulk cells and CSCs. For instance, HIF-1α acts as a pivotal regulator of glycolytic enzymes, including GLUT1, GLUT3, HK, lactate dehydrogenase, and MCT, thereby promoting glycolysis (23, 91, 92). It also downregulates OXPHOS and oxygen intake by inducing PDK, thus destabilizing the TCA cycle (23, 92). HIF-1/PKM2 positive feedback loop also enhances the stimulation of glycolysis mediating proteins, thus enhancing the glycolytic switch of oncogenic cells (93–95). On the contrary, MYC has been seen to increase mitochondrial OXPHOS and ROS, enhancing resistance towards chemotherapy in TNBC (96). Intriguingly it has also been documented that MYC/PGC-1α balance in pancreatic cancers governs metabolophenotype and CSC plasticity (30). Besides, p53 also regulates metabolic changes by inducing several other transcriptional factors and metabolic enzymes. Metabolic enzymes like G6PD, GLUT1, GLUT3, GLUT4, GLS2, ME1, ME2 and PANK1 have been seen to be affected with the expression of p53 (68). It upregulates the expression of SCO2 and downregulates TIGAR, thereby enhancing mitochondrial biogenesis and suppressing glycolysis respectively. Stipulation of mitochondrial respiration by p53 is further influenced by GLS2 where the amino acid metabolism decreases ROS levels thus protecting cell DNA damage. Aerobic glycolysis is inhibited by the expression of TIGAR and repression of glucose transporters 1, 3, and 4, which altogether decrease glucose intake. It is also associated with AMPK and PTEN-mechanisms, further inhibiting the Warburg effect and enhancing gluconeogenesis. Several shreds of evidence have also stated the inhibition of NADPH and glutamine metabolism, lipogenesis and PPP, due to p53 (22, 68).

## Epigenetic regulations of STEMNESS OF CSCs in few cancers

### DNA methylation-demethylation

DNA methylation refers to the process of methylation of cytosine residues of the CpG dinucleotides present in mammalian DNA, using DNMTs – DNMT1, DNMT3A, DNMT 3B. DNMT1 is recognized as a maintenance methyltransferase, which ensures the accuracy of replication of inherited epigenetic patterns. DNMT3A and DNMT 3B are required for *de novo* DNA methylation at unmethylated CpG sites (97). Though DNMTs have distinct roles, they ally to safeguard DNA methylation around the genome loci, viz. CpG islands (CGI) loci are obstinate to *de novo* DNMT3A2 action, which results in DNA methylation (98). However, these DNMT-dependent workouts play a vital role in the regulation of maintaining cellular identity, stemness of a cell, and regulation of CSCs (99). Studies have demonstrated an association between PcG (Polycomb group) targeted methylation in cytogenetically-normal AML (100). A comparative study showed that hypomethylation was reported in a total of 68 differently methylated regions of breast CSCs, like that of non-breast CSCs (101). DMNT3A-knockout derived myeloid carcinomas led to the expansion of preleukemia stem cells, indicating that methylation aberrations induce the growth of CSCs (102). Notably, several pioneer epigenetic changes, including gene silencing and tumor progression, occur due to DNA demethylation. It is the process of removing methyl groups from cytosines of DNAs through the conversion of 5-methyl cytosine to thymine by ten-eleven translocation proteins (TET) and activation-induced deaminase (AID). TET enzymes, TET1 and TET2, promote gene expression of pluripotency genes Oct 4 and facilitate the formation of iPSCs (103).

Oncogenic dysfunction due to DNA methylation is also induced by endogenous metabolite levels and other environmental stress and vice versa (104). Recruitment of DNMTs at the FBP1 promoter was seen to be critical for the expression of FBP1 in BLBCs thus promoting glycolysis (13). Epigenetic inactivation of FBP1 has also observed in HCC, gastric, and colon cancers due to promoter hypermethylation (105, 106). Goel et al. also previewed the significance of methylation-demethylation in the expression of HK2 in tumour infected hepatocytes (107). The demethylation prospective of the Jumonji-C domain-containing Histone demethylases (JHDMs) and TETs also have been seen to be associated with α-ketoglutarate and succinate which further control the TCA cycle (22, 108). Yet another linkage of metabolism with methyl transfer is the usage of S-adenosylmethionine (SAM) as substrates. SAM is spawned from the coupled cycles of methionine and folate, often called one-carbon metabolism. This one-carbon metabolism involves the synthesis of micronutrients like zinc, methionine and the family members of vitamin B and hence is profoundly found active in cancers (109). However, the transfer of the methyl group from SAM to the substrate forms S-adenosyl homocysteine (SAH), which further builds up to the level of inhibiting DNMT and HMT (Histone methyltransferases) activity.

### Histone modifications in CSCs

Histone modifications refer to the alteration of gene expressions *via* acetylation, methylation, ubiquitylation, or phosphorylation (110). These alterations in histone landscapes help establish cellular identity, signifying that they may be involved in acquiring stemness within the cells. In particular, repressive complexes Polycomb group complex 1 and 2 (PRC1 and PRC2) mediated overexpression of EZH2 have been shown to induce tumorigenesis and show relevant characteristics of CSCs in cancers like prostate, breast, PDACs, and different types of lymphomas (111). Several studies have demonstrated that BMI-1 reinforces bivalent histone domains in multipotent progenitor cells and is implicated in the maintenance and invasiveness of cancers like leukemia, GBM, human nasopharyngeal cancer, breast cancer, and endometrial cancer (112–115). Similarly, Brahma-related gene 1 (BRG1) has also shown indispensable aberrations in the Wnt pathway leading to intestinal adenoma, AML, non-small cell lung cancer, breast cancer, and pancreatic cancer (116–120).

Likewise, histone deacetylase (HDAC) 7 also promoted tumorigenesis of the lungs by inhibiting Stat3 (121). Higher levels of HDACs have been associated with advanced tumorigenesis and poor prognosis (122). HDAC7 protein level was overexpressed in human pancreatic cancers, breast CSCs, and CD-10 positive acute lymphoblastic leukemia (123–125). It was determined by Chang et al. that upregulation of HDAC7 was involved in regulating cell proliferation, cell death, differentiation, and metastatic properties, signifying the invasiveness of the protein. It suppresses transcriptional factor MMP10 by mediating myocyte enhancer factor-2 (MEF2), suggesting that HDAC7 regulates vascular permeability (126). HDAC1/7 was associated with CSC-suppressor miR-34a (127), a potential functional signature for the prognosis of glioma and osteosarcoma (128, 129).

Theoretically, active chromatin at promoters can change cellular identity, i.e., de-differentiation and, most importantly, switching to CSCs. A direct association between CSC generation and bivalency was shown in CD44^+^ breast cancers (130), where the response of ZEB1 with TGFβ led to an increase in transcription of ZEB1, which further led to switching of non-CSCs to CSCs. Expression of ZEB1 and ZEB2 have also been associated with glycolytic phosphoglucose isomerase (PGI) in several breast cancer cells (131). Similarly, other bivalent genes are involved in regulating embryonic stem cell differentiation. Polycomb-like 3 (Pcl3) mediates PRC2 in binding to target genes and promotes the expression of pluripotency in ESCs. Pcl3 was also found to be elevated in multiple primary tumor samples viz. cancers of the colon, epithelial, skin, uterus, cervix, and liver. Overexpression of such factors in such cancers can be potentially used for diagnostic purposes (132, 133).

Concerning metabolism, several shreds of evidence suggest the significance of histone modifications (acetylations, methylations, ubiquitylation etc.) in deciding the oncometabolite fate and vice versa. Oncometabolites like 2-hydroxyglutarate (2-HG), fumarate, and succinate accrete in virtue of the mutations in isocitrate dehydrogenases (IDHs), fumarate hydratase, and succinate dehydrogenases respectively, and further induce alterations in the histones and DNAs, which thereby illustrate a metabolic pseudohypoxia, reprogramming the metabolic fate (134, 135). These mutations in IDHs (IDH1 and IDH2) promote tumorigenesis by accumulating a rare oncometabolite D-2-hydroxyglutarate (D2HG). This D2HG further occupies the α-ketoglutarate binding sites, thus inhibiting JHDMs, a major family of enzymes responsible for the regulation of histone methylation. Some reports have also shown the intervention of D2HG with the stability of transcriptional factors HIF1 and HIF2, which further target the hypoxic metabolic pathway, however, the accuracy of the results remains contentious. Mutations in IDH enzymes have also been seen to upset the NADP/NADPH ratio and alter the forward-backward reactions of α-ketoglutarate to isocitrate while consuming NADPH, thus altering the metabolic microenvironment of tumors (136, 137).

### Nucleosome remodeling in CSCs

The basic unit of DNA packaging is the nucleosome, consisting of DNA wrapping eight histone proteins, two each of H2A, H2B, H3, and H4. The presence of nucleosomes usually decides the sensitivity of gene expression in the chromatin, preventing access to the transcription machinery. Hence, nucleosome positioning also plays a vital role in the pluripotency of stem cells and the formation of CSCs (138). Four families of ATP-dependent complexes achieve positioning of a nucleosome, namely switch/sucrose non-fermenting (SWI2/SNF2), imitation switch (ISWI), inositol requiring 80 (INO80), and Mi-2/CHD (139, 140). Aberrant mutations in these complexes, however, have been linked with cancer. Mutations in SWI/SNF and hSNF5/INII have shown a distinct role in the aggression of tumors like malignant rhabdoid tumors, ovarian clear cell carcinoma, gastric cancer, pancreatic cancer, multiple myeloma, breast cancer, GBM, HCC etc (141–160). Likewise, research carried out by Lee et al. showed that INO80 subunits were frequently present in high copy numbers in the colon, suggesting that they promote colon cancer (161). Zhang et al., 2017 defined the role of INO80 in the enhancement of non-small cell lung cancer and revealed a potential therapeutic strategy for inhibiting cancer transcription networks using INO80 (162). Similar results were obtained by Zhou et al., where INO80 governs tumor growth in melanoma (163). It was also found that INO80/NANOG was overexpressed in cervical cancers, suggesting that INO80 can act as a potential therapeutic method for treating cancers (164).

Reprogramming of metabolic cofactors also possesses significant alterations in chromatin remodeling from the aspect of CSCs. Metabolic cofactors like NAD, FAD, PARP, SAM, LSD1, Coenzyme A have shown transformational changes in chromatin structure (54, 165). PGC-1α has been known to be associated with SIRT1 and AMPK to perturb the metabolic stature of cells. In addition, this SIRT1 has been seen to facilitate NAD+ dependent deacetylation of target molecules and orchestrate metabolic agitations by stimulating transcriptional factors like PPARs, p53, FOXO etc. NAD+/NADH ratio also helps in maintaining stemness features, mitochondrial quality, and active aerobic glycolysis, thus highlighting the significance of SIRT1 as a metabolic game-changer (54). Other factors like BAF/PBAF also regulate the energy metabolism pathways in cancer, thereby being a critical regulator of metabolic homeostasis (166).

### Role of miRNA and histone marks in CSCs

miRNAs are small non-coding RNAs that regulate post-transcriptional gene expression by destabilizing the mRNA and translational silencing. These miRNAs have been seen playing a crucial role in maintaining stemness in both normal stem cells and CSCs. Deregulated expressions of miRNAs have been seen to play a critical role in tumor initiation and prognosis. Though genetic modifications in these miRNAs are crucial for initiating tumor suppressor genes, several theories have hypothesized the underlying oncogenic potential of miRNAs associated with the maintenance, growth, and function of CSCs (167). Some signal transduction pathways, such as Wnt/β-catenin, Notch, HH, JAK/STAT, NF-ĸB etc., are often distorted due to the dysregulated expression of miRNAs, further orchestrating carcinogenesis and regulation of CSCs. However, several miRNAs such as miR-134, mirR-296, and miR-470 suppressed the expression of pluripotency maintenance factors Oct4, Sox2 and NANOG. Similarly, let-7 and miR-200 inhibited the expression of Lin 28 and c-Myc, respectively, which were notable self-renewing factors. Further, recent studies about the regulatory functions of miRNAs on CSCs are summarized below.

### Prostate cancer

The aberrant overexpression of miR-1301-3p promotes prostate CSC by inhibiting SFRP1 and GSK3β (168). Further, miR-424 and mir-7 were found upregulated in prostate tumors and featured enhanced cell migration and invasion, increased stemness characteristics, and formation of prostatospheres (169, 170). However, several miRNAs like miR-34a (via CD44), miR-7 (via KLF4/PI3K/Akt/p21 pathway), and miR-320 (via Wnt/β-catenin signaling) inhibit prostate CSC growth reduce stemness and metastatic features. Class III HDACs like SIRT1, is also overexpressed in prostate tumors, where they inactivate other tumor suppressors like HIC1 and activate tumor-promoting genes like p53, N-Myc, cortactin etc. Other histone marks like H3K27 methylation, modification of H4K16, H3K4, H3K56, H3K9, K14, H4K5, H4K12, H3K18, substrates of SIRT1, LSD1, H3R2, H3R42 and EZH2 and mutations in KAT7 and KAT2A are also noted in prostate cancers (170–173).

### Hepatocellular carcinomas

Overexpression of miR-6875-3p, miR-106b-5p (via PTEN/PI3K/Akt pathway), miR-55 (via NF-ĸB), miR-191 (via HIF-2α), miR217 (via DKK1), miR-500a-3p (via negative regulators of SOCS2/SOCS4/PTPN11 of STAT3) and miR-1246 (via Wnt/β-catenin signaling) in HCCs promotes tumorigenesis, stemness and metastasis. Overexpression of miR-137 also induces drug resistance by degrading ANT2 in HCCs. Enhanced stemness markers such as CD90, EpCAM, and Oct4 due to exposure to arsenic also led to the spheroid formation in liver CSCs (174). Negative regulation by miR-612 and CD-133 reduced stemness and metastatic characteristics in HCCs and subsequently inhibited the activity of Sp1 and NANOG (174–183). PAD4 of the partitioning and anchoring domain family, which is known to citrullinate several histones like H2A, H3, H4, and H1R54 was also found to be overexpressed in HCCs, thus contributing to tumorigenesis. Besides, like prostate cancer, modification of H4K16, H3K4, H3K56, substrates of SIRT7 and KMT5A was correlated with HCCs (173, 184, 185).

### Breast cancer

Breast CSCs show up-regulation of miR-29a (basic fibroblast growth factor-induced), miR-137 (via β3/Wnt pathway), and miR-221/222 (via PTEN signaling), signifying that the listed miRNAs are required for the maintenance of the CSCs. However, unlike other cancers, up-regulation of other miRNAs inhibited cell proliferation and induced apoptosis in triple-negative breast cancer and breast CSCs. MiRNAs like miR-1287-5p, miR-27a, miR-34a, miR-628, miR-142-3p, miR Let-7, and others were found to reduce tumor growth, potentiate the effectiveness of therapeutic therapies and modulate signaling pathways, thereby inhibiting the renewal of CSCs (186–194). In fact, class I HDAC1 like H4K16 and H3K56, HDAC2, class III HDACs like SIRT1, SIRT2, SIRT3 and SIRT7, HDAC5 (p53), HDAC6 (HSP90 and cortactin), HDAC7 and HDAC11 overexpress themselves in several breast carcinomas. Modification levels of H3K9, H3K18, H4K12, H4K16, H3K4, H4K20, and H4R3 also were related to breast tumorigenesis (185). Histone marks like KAT2A, KAT4, KAT7, KAT6A, LSD1, JMJD2B, JMJD2C, p300, SETD2, SMYD3, and substrates of EZH2 and PAD4 were also seen to be mutated in breast cancer (173).

### Lung cancer

Poor regulation of miRNAs plays a vital role in cellular apoptosis, proliferation, inflammatory response, maintenance of stemness, and metastasis development of the lungs. Associated with self-renewal of lung CSCs and tumor differentiation, micro RNAs like miR-5100, miR-494-3p, miR-19a/19b, and miR-1246 showed upregulation (195–198). Shi et al. showed that miR-34a demonstrated negative regulation of tumor properties in CD44^hi^ lung CSCs and non-small cell lung cancer (NSCLC) (199). Aberrant regulation of miR-218 overexpressed stemness features and IL-6/JAK-STAT3 signaling in ALDH-positive lungs (200). Downregulation of tumor suppressor miR-128 showed tumor differentiation and metastasis by targeting ERK/AKT and p38 signaling pathways (201). Alike HCCs and breast cancer, lung cancers (esp NSCLCs) show upregulated expression of PAD4, compared to their normal or benign hyperplastic tissues (184). Other than that, mutations in KAT2A, KAT2B, KAT4, KAT6A, KAT6B, KAT7, and MYST1are quite prevalent in lung cancers. Though HDAC1 Class I and HDAC 2 display an overexpressed state in lung tumors, lower expression of HDAC9 and HDAC10 are linked to poor prognosis in the same. Histone targets like H3K9, H3K36, and H4K20 have also been seen to be associated with several lines of lung cancer (173).

### Pancreatic cancer

Several reports revealed the role of miRNAs associated with the maintenance of stemness, cancer invasiveness, and metastasis of pancreatic CSCs and PDAC is quite crucial. Overexpression of miR-30a, -30b, and -30c in CD133+ pancreatic CSCs show high metastatic features with increased mesenchymal phenotype markers (202). Dysregulated expressions of miR-744 are often related to Wnt/β-catenin signaling, one of the main pathways of pancreatic CSCs. Upregulation of miR-744 and downregulation of miR-200 potentiates stemness features and drug resistance of pancreatic CSCs (203, 204). HDAC1 substrates like H4K16, H3K56, and HDAC7 substrates also show an overpowered expression in pancreatic cancers, which further leads to poor survival. Low levels of SIRT7 substrate H3K18ac also display a poor prognosis in PDACs, thus contributing to the tumorigenesis of pancreatic cancers (173).

### Metaboepigenetic regulation of pro/anti-tumor immune response

CSCs are endowed with various biological characteristics, which reflect the heterogeneity of these stem cells. However, to promote and maintain tumorigenesis, many a time, these characteristics also play an interdisciplinary role within each other. The immune system’s role which has been primarily attributed towards eliminating CSCs, plays a dual role in both promoting and eliminating cancer progression, suggesting CSC’s enhanced immunoediting mechanisms. This enhanced immunoediting mechanism is primarily regulated by several metabolic and epigenetic factors which decide the fate of the immune responses, thereby promoting cancer progression. Playing as major hallmarks of immune cell phenotype, both metabolism and epigenetics have displayed several pieces of evidence in reinstating tumorigenesis and metastasis in CSCs. In virtue of these factors, tumor cells induce changes in the immune microenvironment by modulating innate immune cells like macrophages, MDSCs, and Tregs and transforming the environment into an immunosuppressive one. In this part of the review, interactions of CSCs, the oncometabolite, and the epigenetic factors with the immune system will be discussed, as well as we will highlight the therapeutic potential of targeting immune cell interactions to invade CSCs. Possible interactions have been summarized below and in **Table 1**.

**Table 1:** A summarized interaction between CSCs and the immune system.

Immune cell	Function		Affected tumor models	Reference
	Effect of immune cells on CSCs	Effect of CSCs on immune cells		
Macrophages/TAM	Promote drug resistance, drive EMT, maintain tumorigenesis	Development of macrophage polarisation towards M2 immunosuppressive phenotype and inhibits macrophages phagocytic and anti-tumor activities	AML, CRC, lung cancer, breast cancer, PDAC, GBM, HCC	(205–207)
MDSCs	Induce the expression of stemness genes and increases EMT	Recruit MDSCs for CSC maintenance	Breast cancer, Lung cancer, lymphomas, renal cell carcinomas, colon cancer, head and neck squamous cell carcinoma, non-small cell lung cancer	(205, 207, 208)
Tregs	Promote the development of CSCs, induce EMT indirectly	Impair the proliferation of effector T cells and promotes the expansion of more Tregs	Non-small cell lung cancer, ovarian cancer, skin tumors, gastric cancer, GBM, breast cancer,	(205, 207, 209, 210)
Neutrophils		Recruit neutrophils to metastasise CSCs	Renal cell carcinoma, melanoma, CRC, T- cell lymphoma, head and neck squamous cell carcinoma, pancreatic cancers, leukemia and Gliomas	(205, 207, 211)

### Macrophages

Macrophages are notable leukocytes that have roles in tissue homeostasis and immunity. Depending upon the tumor microenvironment (TME), these macrophages, also referred to as TAMs, exhibit pro (M1 macrophage) and anti-tumor (M2 macrophage) characteristics (212–214). Several studies have reported the significance of TAMs in mediating immune-stimulatory agents and the destruction of tumor bulks. On the contrary, there is also evidence where they have been seen to participate in tumorigenesis, promoting cancer motility and metastasis of CSCs (215–217). The diversity of such functions is governed in response to local signals (chemokines and cytokines). Though TAMs mostly show an M2- like phenotype, a high M1/M2 ratio is tried to maintain so as to suppress cancer entities. These functional changes also are dependent upon the underlying metabolic changes, thus specifying the complex relationships of metabolic and functional reprogramming of macrophages.

In the early stages of tumors, TAMs produce cytokines like milk fat globule epidermal growth factor (MFG-E8) and IL-6, which activate several signaling molecules and pathways in CSCs, like STAT3 and Hedgehog, thereby mediating DNA damage, cancer-related inflammations, and drug resistance (218). They further modulate CSC plasticity by producing factors like TNF-α, TGF-β1, which is supported by other stromal collagen fibers and stemness factors like IL-8 and CXCL12 (8). Hypoxic conditions also favor CSC angiogenesis and metastasis, as hypoxic factors like HIF-1α and HIF-2α promote VEGF-A production, a major proangiogenic cytokine (219).

However, the M1 macrophages try to restrain tumor growth by releasing type 1 pro-inflammatory cytokines. By upregulating IFN-γ, they characterize the secretion of several ROS and nitrogen intermediates, major histocompatibility complex class II molecules, and other immune-stimulatory cytokines such as IL-12, IL-23, CXCL9, and CXCL10 in CSC niches. These, in turn, drive the recruitment of T_H_1 cells, thereby amplifying a type 1 response. This is further supplemented by a shift in iron homeostasis, which contributes to bacteriostatic effects (220). In relation to metabolism, Liu et al. in their experiment comprehended a similar analysis on tumor-extract stimulated bone-marrow-derived macrophages (TES-TAMs), and found an upregulated aerobic glycolysis. It exhibited a blend of M1/M2 phenotype with upregulation of HK2 and other cytokines like ARG1, IL4Rα, and PLIN2 (221). An upregulation of cytokine production and aerobic glycolysis was also discovered by Arts et al. using immunohistochemistry, showing enhanced expressions of PFKFB3 and PKM2 in TAMs of thyroid carcinomas (222). It was also further supported by Penny et al. when they inhibited HK2 with 2DG and showed disruption in prometastatic phenotype in PDAC cell lines (223). REDD1/mTOR axis was also seen to be upregulated in hypoxic TAMs leading to suppression of glycolysis and increased angiogenic responses (224), thus potentiating the fact that the general preference of TAMs is aerobic glycolysis and further metastasis is promoted and regulated by the expression of HIF-1α/mTOR. This regulated expression also further induces an elevated ARG1 and VEGF expression, which also plays a pivotal role in tumor progression using the ARG1-dependent polyamine synthesis pathway, thus enhancing the polarization of the macrophages towards an M2-like phenotype. Several shreds of evidence have also stated the significance of FA metabolism in influencing the tumoricidal and immunogenic functions of TAMs, viz. overexpressed prostaglandin production, activation of PPARβ/γ *via* IL-4/STAT6 pathway, fatty acid accumulation, and redistribution (225–228).

Concerning the epigenetic modifications relevant to TAMs, several reports have reported the switch of the M2 phenotype to M1 due to these changes, thereby re-polarizing an anti-tumor activity. DNMT3B, TET2, H3K4 methyltransferase, SMYD3, HDAC9 are some of the speculated factors known to be associated with M2 polarization. In fact, this TAM polarization is also associated with TLR4 ligands and LPS, which result in a shift towards glycolysis and impaired mitochondrial biogenesis. The glycolytic shift is accompanied by an increase in lactate which inhibits class II-HDACs. As mentioned above, TET and JHDM, the two crucial oncometabolite also serve as vital metabolic switches for M1/M2 macrophages. STAT6/PPARγ/PGC-1α and the mTORC2-IRF4 signaling axes also play a pivotal role in the regulation of OXPHOS and mitochondrial biogenesis, thus modulating the polarization of TAMs (229). Besides, dysregulation of NF-ĸB signaling in ovarian cancers, overexpression of miR155 in Lewis lung carcinomas, and CSF-1R inhibition in gliomas have shown robust regression of tumor bulks, thereby potentiating epigenetics as a therapeutic opportunity in treating cancers (230–232).

### Myeloid-derived suppressor cells

MDSCs are a subset of immature granulocytes which exert an immune-suppressive phenotype in the CSC niche. They have been seen to suppress NK cells, natural killer T cells, and other T-cell responses, thereby modulating the innate immune system and releasing reactive nitrogen and oxygen species (233–235). Besides, CSCs produce VEGF and SDF-1 to recruit MDSCs into the CSC niche. Tumor-induced chemokines like CCL2, CCL15, CXCL5, and CXCL12 also mediate MDSC recruitment, which further suppresses the immune function, thereby facilitating CSC maintenance (205).

Several evidence has been reported indicating that MDSCs induce stemness genes and drive the EMT in CSCs. They have also been seen to express a dynamic metabolic flux, with increased carbon metabolism, i.e. enhanced glycolysis, PPP and TCA cycle. Owing to the high glycolysis and glutaminolysis rates, several pathways like the PI3K/AKT/mTOR, LXR, PPARγ, AMPK, STAT, PGE2 pathways support cellular proliferation for the synthesis of glucose, fatty acids, proteins, and nucleic acids. Under hypoxic conditions, the mTOR pathway activates HIF-1α (236, 237), which further stimulates enhanced glucose, lactate transporters, glycolytic enzymes, and a repressed OXPHOS, thus mediating the switch from OXPHOS to glycolysis in MDSCs. Besides, carbon metabolism, MDSCs also show an upregulated expression of FA transport proteins, FA translocase CD36, CPT1 and CPT3, thus suggesting FAO as one of the generic fuels for MDSCs (238, 239). G-MDSCs in certain tumors express higher levels of Arg1, whereas M-MDSCs express an overexpressed iNOS to catabolize Arg1 to inhibit MDSC functions, thus associating amino acids with MDSC metabolism (240).

Epigenetic modifications also have been seen to result in a heritable regulation of MDSCs leading to the reframing of the tumor microenvironment of several cancer species. In addition, to support tumor growth and progression, they have been seen to stimulate factors like VEGF, β-EGF, MMP9, and other angiogenesis factors (241–243). Higher expressions of Arg1 and STAT3 have led to the accumulation of MDSCs in cancer models, thus suggesting their tumor-suppressive functions. In a study by Sahakian et al., HDAC11 was noted to be a regulator of MDSC maturation, thus indicating its role as a gatekeeper in myeloid differentiation (244). Concerning microRNA interference, miR-210 enhances the expression of CXCL12, IL-16, Arg-1 in MDSCs, thereby establishing a link between microRNAs and MDSC mediated immune suppression. The expression of miR-101 in ovarian cancer cells and prostaglandin E2 in cervical cancer cells is mediated by MDSCs, thus demonstrating their role in tumorigenesis and metastasis (245, 246). Other microRNAs like miR-9, miR-690, miR-155, miR-21, miR-223, miR-34a, miR-146a, miR-424, miR-181b, miR-17-5p, and miR-20a has been associated with MDSC development and differentiation, and if regulated can promote overall tumor immunity (244). In addition, MDSCs have been seen to foster STAT3 signaling in breast CSCs and PDACs by inducing stemness factors like IL-6 and nitric oxide (NO), which subsequently increase ALDH1^+^ CSCs (247, 248).

### Regulatory T Cells

T regulatory cells (Tregs) orchestrate the function and activation of other immune cells and play a crucial role in controlling T cell-mediated autoimmunity (249). In regard to cancer, CSCs alter the immune landscape and promote the expansion of immunosuppressive and pro-tumorigenic Tregs. Several studies have shown that CSCs from glioblastoma, breast cancer, prostate cancer, mesothelioma cell lines, and head and neck squamous cell carcinoma impairs the production of effector T cells and stimulates Tregs production (250–253). CSCs also produce TGF-β pathway members, which act in concert with the regulation of Tregs. With increased tumor formation, this TGF-β inhibits the accumulation of CD8+ T cells and secrete inflammatory cytokines such as IFN-γ, TNF, IL-6, and CCL2, thereby maintaining stemness in CSCs (254).

On the contrary, Tregs also produce TGF-β, which promotes invasion, angiogenesis and drives the epithelial-to-mesenchymal transition (EMT) process (255). Furthermore, Tregs also influence the expression of programmed cell death 1 on CD8^+^ T cells and enhance VEGF levels in the presence of hypoxia, which promotes angiogenesis, thereby affecting the stemness and progression of CSCs (256, 257). In addition, recent studies have shown that FOXP3+ Tregs induce IL-17, which under a hypoxic condition, potentiates CSC development in CRCs. Further results showed that IL-17 acts in a STAT3 dependent manner (258).

Expression of FOXP3 is also mediated by notable metabolic factors like PPARγ, HIF-1α, and mTOR and PD-L1, which are maintained by the balance between glycolysis and FAO in Tregs, thus caveating the control of metabolism over differentiation of Tregs (228, 259–261). Depending upon the tumor microenvironment, Tregs avail alternate substrates for their metabolic sustenance. In steady states, they are known to display an enhanced glycolytic rate and lipid biosynthesis with the hyperactivation of mTOR. During proliferation, they increase the expression of GLUT1, activate mTORC1, and display a comparatively higher glycolytic activity, thereby suggesting that Tregs are the most enthusiastically proliferating compartment *in vivo* (262–265). However, deletion of PGC-1α and SIRT3 abrogated Tregs suppressive function both *in vivo* and *in vitro*, hence suggesting the role of OXPHOS in the proliferation of Tregs (266, 267). Yet, another interesting theory also suggests the engagement of epigenetics in the differentiation program of Tregs. The role of Enolase/MYC Binding Protein-1α also has been associated with the expression of glycolysis and FOXP3 splicing variants (268). Suppression of EZH2 in the tumor microenvironment also has shown sustained generation of Tregs from naïve precursors, thus potentiating tumor growth (269). Evidence has also stated that enhanced expression of tryptophan metabolizing enzyme IDO also leads to increased Treg cell infiltration in several cancers like CRC, HCC, and cervical cancers, and also has been seen to increase the frequency of metastasis in these cancer lines (267).

### Neutrophils

Neutrophils account for the maximum amount of leukocytes in the human blood. Thus, apart from eliminating pathogens and maintaining a circuit of adaptive immunity, neutrophils constitute a vital portion of the immune cells invading the CSC niche. Several studies have demonstrated the role of TANs in cancer progression and metastasis. However, similar to TAMs, TANs also show a cytokine-driven polarisation wherein they can be modulated into a pro- (N2) or an anti- (N1) tumorigenic phenotype.

The extent of neutrophil infiltration was suggested by Hira et al. when they examined glioma stem-like cells in high-grade glioma patients (270). A similar prognosis was also obtained in cases of aggressive forms of renal cell carcinoma, melanoma, CRC, head and neck cancer, pancreatic neoplasia, and micro-papillary carcinomas (271, 272). TANs have also been shown to facilitate metastasis by suppressing NK-cell mediated clearance of tumor cells and recruiting TAMs at inflammatory sites through the secretion of IL-8, TNF-α and myeloperoxidase (273, 274). However, in the presence of type-1 IFNs, TANs display an antitumor N1 phenotype with increased tumor cytotoxicity and escalated levels of ICAM1, TNF-α, and NET (neutrophil extracellular trap) (275).. In addition, there were upregulations of pro-inflammatory cytokines and chemokines like IL-12, GM-CSF, CXCL10, CCL7, CCL2, and CCL3 in the N1 phenotype. This cytokine expression promotes CD8^+^ T cell recruitment and activation, thereby potentiating the antitumor effect, thence suppressing tumor growth (211).

Metabolically, TANs like other immune cells strongly rely on aerobic glycolysis and PPP as their dominant form of metabolism. Mitochondrial action also progressively diminishes as neutrophils mature, as they lose their c-kit expressions; however immature neutrophils and glucose-restricted tumor microenvironment rely on mitochondrial biogenesis, thereby maintaining a local immune suppression and ROS production (276). This comes in line with the fact that NADPH, an important cofactor for NADPH oxidase is produced by the PPP pathway, also stimulates neutrophil microbicidal functions. Yet, another function of neutrophils, the formation of neutrophil extracellular traps (NETs), a mixture of several epigenetic elements, i.e. DNA, histones, antimicrobial-peptides; also works in virtue of the metabolic shift towards glycolysis, glucose uptake, and PPP (277, 278). NETs also have been associated with several metastatic factors and other proinflammatory adhesion molecules, which further contribute to cancer-stimulated organ failures, thus demonstrating the characterization of NETs towards tumor progression (228, 279).

## Therapeutic advances

### Targeting CSCs *via* metabolism

Classically, the metabolism signature of CSCs is quite different from that of regular cancer cells, which offers novel opportunities to target CSCs specifically. Several approaches have been made targeting glycolysis, mitochondrial biogenesis, OXPHOS, PPP, and FAO.

Drugs have been developed targeting glycolysis to suppress GLUT1 and regulate pyruvate metabolism. Glycolysis blockers along with competitive inhibition of phosphoglucoisomerase induced cell death in human CD44^+^/CD24^low^ breast CSCs (only, not their non-CSC equivalent) (280). Similar approaches were tried with prostate CSCs, which resulted in induced cell death, reduced tumor spheroid formation, and higher dependency on glutamine, which can further be treated with glutaminase blockers (281). GLUT1 inhibition *in vivo*, by administration of WZB117, inhibited tumor initiation by CD133^+^ affiliated CSCs (glioma, pancreatic and ovarian CSCs) (282). Experiments by Li et al., and Isayev et al., extended the knowledge about anti-cancerous properties of 3-bromopyruvate (3BrP) (51, 283). 3BrP is known to suppress the cell viability of clonogenic RPMI8226 myeloma cells, KG1 leukemia cells, HepG2 hepatoma cells, and DU145 prostate cancer cell line by inhibiting ATP-dependent efflux pumps (284). Yet, another promising candidate in this field turned out to be metformin, an anti-diabetic drug. Compelling evidence showed that metformin induces an energy crisis by inhibiting mitochondrial complex I and impairing the ability to switch to glycolysis, subjecting to cell death (30, 285).

Suppression of mitochondrial OXPHOS by blocking ETC complexes also helps in modulating CSC populations (30). Treatment with oligomycin targets mitochondrial H^+^-ATP synthase and has shown commendable results in treating pancreatic, CD44^+^/CD117^+^ ovarian, and CD87^+^ lung CSCs and their non-tumor bulk variant too (29, 281, 286). SLC25A10 inhibition in combination with radiation therapy has shown also significant improvements in treating cancers, especially solid tumors with hypoxic cell fractions (40).

Considering the importance of lipid metabolism in CSCs, drugs intervening in different aspects of fatty acid metabolism have yielded positive results. Inhibition of FASN has showed suppressed tumor growth in brain CSCs, CD24-/CD44+/ESA+ ovarian CSCs, and breast CSCs (287, 288).

Intriguingly, mTORC1 activation leads to glutamine anaplerosis by activating glutamate dehydrogenase. On the other hand, mTORC1 downregulates SIRT4, which is a critical negative regulator for glutamine metabolism. However, mTOR inhibitors like rapamycin targets show efficacy in deregulating the mTOR pathway, thereby destabilizing the glutamine metabolism of the CSCs (289). These mTOR inhibitors have shown potency in reducing angiomyolipomas’ tumor growth in clinical trials of tuberous sclerosis (290).

Recent studies have also shown that combined therapy of chemotherapeutic drugs and Vitamin C induces an effective treatment mechanism. Vit. C interferes with the metabolic interests of the CSCs and also disturbs the epigenome regulation in them (291). Further research should focus on using Vit. C as a viable source to facilitate cancer treatment. Furthermore, evaluating a combined drug-controlled process aiming to destroy metabolic adaptability would condemn CSC survival.

### Targeting CSCs *via* epigenetics and metabolic blocks

As a whole, several characteristic metabolic, genetic, and epigenetic signatures can act as biomarkers for detecting CSCs. Over the past couple of decades, numerous methods have been envisaged regarding the dysregulation of cell signaling or cell surface proteins to treat CSCs.

DNMT inhibitors were among the first epigenetic therapeutic molecules which were incorporated for cancer treatment. Low doses of these inhibitors show profound efficacy in decreasing DNA methylation, thereby reducing tumorigenesis. Transient exposure to such low-dosed inhibitors induced antitumor response and decreased stemness properties in primary leukemia and epithelial tumor cells. DNMT inhibitors like decitabine and azacitidine have been broadly used to treat cancers of the breast, colon, and lungs. They have also shown significant downregulation of stemness factors like NANOG and Oct4 in prostate cancer (292, 293). Knockdown of DNMT1 decreased IL-6 mediated lung tumorigenesis and cancer stemness (294). Administration of SGI-110, along with other chemotherapeutic drugs, has repressed the stemness properties of ALDH+ ovarian cancer cells and inhibited tumorigenesis (295). Clinical trials with SGI-110 are still ongoing to treat various other CSCs like Liver, Leukemia, Ovarian, etc.

Yet, another procedure for treating CSCs is targeting histone modifications, like HDAC, LSD1 inhibitors etc. LSD1 or Lysine-specific demethylase 1A mediates transcriptional activation/repression, thereby enhancing proliferation, invasiveness, and cell motility. However, effective LSD1inhibitors and their corresponding analogs have shown significant antitumor activities across solid tumors and CSCs (296). HDACs have also been known to remove additional acetyl groups, thereby stimulating gene expressions. HDAC inhibitors like vorinostat and romidepsin have been approved to treat cutaneous T-cell lymphomas. HDAC inhibitors have also been shown to repress stemness in certain CSCs by reprogramming cancer cells. Gottlicher et al. demonstrated that valproic acid, a potent HDAC inhibitor known to be an antiepileptic drug, relieves HDAC mediated transcriptional repression, which further induces apoptosis of carcinoma cells, hematopoietic stem cells, and leukemia blasts from AML patients. This was further amended by Travaglini et al., that valproic acid reprograms the malignant mammary epithelial cells to a more physiologic phenotype and improves the sensitivity towards chemotherapic treatment (297, 298). Yet, another inhibitor of class I HDACs, entinostat, has been shown to decrease tumor-initiating cells from triple-negative breast cancer, hence reducing expression of several stemness factors like BMI-1, NANOG, and Oct-4. They have also led to prevent tumor formation at primary sites and distant metastasis (299).

Similarly, miRNAs also play a crucial role in regulating CSCs, which can become therapeutic tools when exploited. MiR-34a has been associated with CSCs of the prostate and pancreas, which, when supplemented with inhibitors of DNMTs and HDACs, lead to inhibition of stem cell proliferation and metastasis (172, 300). Suppression of miR-200c and miR-9 were found to be associated with the treatment of breast CSCs and inhibiting tumor formation in the breast (301, 302). MiR-126, when inhibited, led to the eradication of leukemic cells in AML (303). Combinations of anti-miRNA nucleotides and chemotherapeutic drug Sunitinib have shown anticancer effect in PDAC, indicating that miRNA inhibition therapies, when complemented with chemotherapeutic drugs, suppress stemness markers and stem cell properties of the tumor (304).

### Targeting CSCs *via* immunotherapy

As discussed above, it can be postulated that CSCs can shape the immune system to harness their own needs. Vice versa, the infiltrating immune cells interact with CSCs change their immunogenic signature, and promote stemness, tumorigenesis, and metastasis of CSCs. However, the review also mentions the anti-tumorigenic potential of M1-TAMs and N1-TANs, and if appropriately modulated, might improve the clinical outcome of the patients. However, as mentioned earlier, immune cell-CSC interactions, including M2-TAMs, N2-TAMs, Tregs, and MDSCs, can be fruitful therapeutic structures if reversed using signaling techniques. Possible therapeutic strategies have been summarized in **Table 2**.

**Table 2:** A summarized form of immunotherapies (*CT = Combinational therapy).

Immunotherapies	Type of immunotherapy	Effective CSC/tumor models	References
Innate Immune response	NK cells	Glioma CSCs, Colorectal CSCs, Oral Squamous Carcinoma CSCs	(305–307)
	γδ T cells of the Vγ9/Vδ2 phenotype	Colon CSCs, Ovarian CSCs	(308, 309)
	Zoledronate-activated γδ cells (CT)	Breast CSCs, Ovarian cancer, Melanoma, Colon Cancer, Cervical cancer	(310)
CSC vaccines	DC vaccination	Breast CSC, Prostrate CSC, Lung metastasis, Melanoma, Squamous cell carcinoma	(311–314)
	DNA vaccination	CRPC, Renal Cell Carcinoma, Lung	(315–318)
T cell-based immunotherapy	CSC-primed T cells	Breast CSC, Head and Neck squamous cell carcinoma, Pancreatic CSC, Lung CSC	(319, 320)
	CSC-CAR T cells	Melanomas, TNBCs, GBMs, Head and Neck squamous cell carcinoma, Sarcoma, Mesothelioma, Gliomas, Prostate CSC	(321–325)
mAb	Anti-CSC marker-based mAb	Breast cancer, melanoma, hepatocellular CSC, pancreatic CSC	(326–328)
	HER2-targeting mAb (CT)	Breast CSC, GBM	(329–331)

### Innate immune response to CSCs

As frontline workers of the immune system, the natural killer (NK) cells constitute the primary lymphocytes responsible for invading tumor cells. However, regarding CSCs, the role of NK cells remains quite contentious. For example, Wu et al. suggested that CD133+ and CD133- cells in brain CSCs adopt a mechanism to escape immune response mediated by MHC1 or NK cell-activating ligands, thereby making them obstinate to the innate or adaptive immune monitoring. Further, treatment of CD133+ cells with IFN-γ renders them sensitive to NK cells *in vitro* (332). Similar observations were reported in the case of breast CSCs, where CSCs dodge NK cell-mediated immunity due to downregulation of MICA and MICB (MHC I-related chain A and B), the two ligands for the stimulatory NK cell receptor NKG2D due to aberrant expression of miR-20a (333).

However, conversely, certain CSCs like glioma, colorectal and oral squamous carcinoma have expressed a significant amount of NK cell receptors, thereby mediating NK cell toxicity (305–307). These conflicting results thereby help us determine that CSCs in different tumor systems foster different sensitivities.

Yet another mode of therapy is done by γδ T cells of the Vγ9/Vδ2 phenotype, a part of the innate immune family. Human Vγ9/Vδ2 cells have been seen to be effective against colon CSCs, ovarian CSCs, and neuroblastoma. Zoledronate-activated γ cells showed positive responses against breast CSCs, solid tumors like ovarian, melanoma, colon, and cervical and enhanced the potential of CD8^+^ T cells *via* the IFN-γ driven overexpression of MHC I and ICAM 1 (308–310).

### T-cell based immunotherapy

T-cell-based immunotherapy entirely relies on the production of effector T cells, accompanied by the adoptive transfer of CD8^+^ T cells back into patients. For CSC-primed T cells, CSCs derived from carcinoma cell lines like breast, head, and neck, and pancreas could induce a CD8^+^ T cell response. Adoptive therapy with ALDH-specific CD8^+^ T cells could be used to kill ALDH^hi^ CSCs *in vitro*, thereby suppressing tumor growth and metastasis (319). Similar techniques were adopted by Luo et al. when they used CSC lysate-pulsed dendritic cells to stimulate CD8^+^ T cells to inhibit lung CSCs. Their study showed that ALDH^hi^-CD8^+^ T cells mediated antitumor effects, thereby suppressing tumorigenesis and metastasis (320).

Nevertheless, CAR T cells also symbolized to be successful immunotherapy. Using ex vivo genetic crafting, T cells can be genetically engineered to manifest a T cell receptor or CAR to recognize TAAs and inhibit tumor growth. CSC antigens like CSPG4 in melanomas, TNBCs, GBMs, head and neck squamous cell carcinoma, sarcoma and mesothelioma (321), CD 133 in GBMs (322), EGFRvIII and IL13Rα2 in gliomas (323, 324), and EpCAM in prostate carcinomas (325), are used as targets for CAR T-cell based immunotherapies. Neoantigens, which can be identified efficiently by T cells, also show promising CAR T cell therapy candidacy.

### Monoclonal antibody-based immunotherapy

mAb-based immunotherapy has been considered a successful cancer therapy in the 21^st^ century. Immunomodulatory drugs, demonstrated by anti-PD-1, anti-PD-L1, and immune checkpoints targeting anti-CTLA-4 antibodies, have shown profound success in clinical trials against cancer. The expression levels of CSC markers also act as attractive targets for antibody-based immunotherapy. For, e.g., anti-CD44 mAb inhibited tumorigenesis of murine breast tumors and showed regression in human melanoma metastasis (326, 327). CIK cells bound with anti-CD3/anti-CD133 bispecific antibodies were used to inhibit the growth of CD133^hi^ CSCs of the liver and pancreas (328).

HER2-targeting antibodies, like trastuzumab and pertuzumab, also dramatically reduced CSC populations in GBMs and Breast CSCs. However, as CSC markers are not genuinely defined for specific subsets of CSCs, single mAb therapy may cause side effects to neighboring normal cells. Moreover, combinational therapy using mAb cocktails can efficiently eradicate CSC populations while reducing the side effects to other normal cells (329–331).

### CSC based vaccines

Immunotherapeutic strategies are based on the endogenous activation of T-cell responses to malignant cells *via* administering tumor-associated antigens (TAAs) to patients, which is successfully performed using vaccines (Dendritic cell, peptide, whole-cell or genetic). Specifically, the DC-mediated tumor immune response is stimulated by CSCs, which act as antigen sources (334, 335). However, it has been observed that enriched immunogenic CSCs are more effective than their non-stem counterpart. Administration of dendritic ell vaccines to syngeneic tumor models of immunocompetent mice has led to a substantial decrease in lung metastasis, squamous carcinoma cells, prostate CSCs, and regression in tumor models of melanoma D5 and squamous cancer SCC7 (311–313). Breast CSC-Dendritic Cell vaccines also showed a CTL-antitumor response by stimulating CD8^+^ and CD45^+^ T cells (314).

On the contrary, DNA vaccinations have been used as a new strategy to target cancers. They involve injecting genetically engineered plasmids for direct production of antigens, leading to a culminating immune response, thereby preventing cancer. Albeit in clinical trials, several DNA-based vaccines have successfully elicited antigen-specific T cell immune responses. In human castration-resistant prostate cancer, both PAP (prostate acid phosphatase) and PSA (prostate-specific antigen) is administered using DNA-based vaccinations (315, 316). Immunization with CSC-specific DNAJB8 expression plasmids also showed commendable anti-tumor response compared to immunization with TAA survivin (expressed in renal cell carcinomas) (317). Polakova et al., in their experiment, also used DNA vaccinations to induce antitumor effect against mouse oncogenic Sox2-expressing lung TC-1/B7 tumor cells (318). In sum, it can be said that CSC-DNA vaccines hold great potential to serve as a tool for immunotherapy, and they will turn out to become a great asset in cancer therapy in the years to come.

CSCs are regulated *via* three perspectives, namely metabolism, epigenetics, and immunology. It has been identified that CSCs acknowledge various phenotypes of metabolic reprogramming, the glycolytic pathway, oxidative phosphorylation, mitochondrial biogenesis, glutamine metabolism, Pentose Phosphate Pathway etc. Epigenetic dysregulation of CSC-related signaling pathways, i.e. Wnt, Notch, and Hedgehog leads to self-renewal ability and therapeutic resistance. Immune cell-CSC interactions have also been seen to alter the functional and genetic makeup of both the immune cell subsets and CSCs, leading to impaired CSC recognition and elimination by the immune system. With several processes in hand, CSCs evolve with varied niches and render their lifestyle procedures efficiently. (HK= Hexokinase, LDH= Lactate dehydrogenase, PK= Pyruvate kinase, PDK= Pyruvate dehydrogenase kinase, G6PDH= Glucose-6-phosphate dehydrogenase, SCD= Stearoyl-CoA, FASN= Fatty Acid synthase, GDH= Glutamate dehydrogenase, GLS= Glutaminase, VEGF= Vascular endothelial growth factor) (Created with BioRender.com)

### Diagnostic and blood-based biomarkers: Metabolic and epigenetic profiling for early cancer detection and treatment outcome

Manifesting properties like self-renewal and stemness, metabolic alterations, epigenetic modifications, and immunogenic rewiring, cancer cells and their stem cell analogous can be considered different from naïve cells. With varied such properties, they characterize themselves with several markers, which are also known as biomarkers. Facilitating with the molecular definition of tumor, these biomarkers are subject to dynamic modulation and are expected to enhance our understanding of tumor metabolism, epigenetic and immunogenic influence. This would further provide insights into new therapeutic approaches for eradicating CSCs and their tumor bulks.

Enhanced carbon metabolism also plays a prominent and fundamental change in many cancers irrespective of their nature of origin or subsequent histological changes, thus entailing a great potential as cancer biomarkers. Enhanced glucose uptake using GLUTs and HK2 levels (336) has been observed to be efficient candidates for conferring the glycolysis metabolism inside tumors. Mutations in IDH enzymes also implicate metabolic rewiring, thus providing references to tumors (337). Positron emission tomography (PET) metabolism biomarkers like ^18^F-fluorodeoxyglucose, ^18^F-fluoroethyltyrosine, ^18^F-dihydroxyphenylalanine, ^18^F-fluorothymidine, and other radiotracers like ^13^N-ammonia, ^15^O-water, ^82^Rb-chloride, ^11^C-palmitate, ^11^C-glucose, ^11^C-acetate, etc. are also associated with perfusion and metabolic index of specific cancers (338).

Cancer, also being a cluster of alterations, confers a broad spectrum of genetic/epigenetic alterations, mutations, gene amplification, accompanied by varied methylation and acetylation changes. With DNA hypo- and hyper-methylation being the apparent epigenetic events responsible for promoting cancer, several studies have also shown the involvement of DNMTs in developing abnormalities in naïve cells. Blood/serum gene panels have shown promoter hypermethylation of several genes like GSTP1/TIG1/PTGS2/RPRM for prostate carcinomas (339), APC, RARβ2, RASSF1A, and p16/INK4a for lung cancers (340, 341), ITIH5, RASSF1A, and DKK3 for breast cancers (342), MLH1, VIM, TFPI2/HPP1 and APC for CRCs (343–345), MGMT, RASSF1A, p15/INK4b and p14/ARF for gliomas (346), etc. Concerning histone modifications, H3K4, H3K9, H3 and H4 in prostate cancer, H4K20 in preneoplastic lesions, H2A in breast cancer, H3K27 in midline gliomas are some of the notable expressions associated with early cancer detections (347). MicroRNAs and lncRNAs also play a critical role in detecting tumors and have already been described in the review. Immuno-specific antigen-based biomarkers like prostate-specific antigens, alpha-foetoprotein, cancer antigens, carcinoembryonic antigen, human chorionic gonadotrophin (hCG), HSPs, TGF-β, VEGF, EGFR, YKL, and MMPs are also seen to be expressed in many malignant tumors (339, 348).

However, to date, identifying markers to specific CSCs remains a challenge, as most of the CSC biomarkers label a heterogeneous stem cell population. However, combinations of extracellular and intracellular markers give prominence towards the isolation and characterization of specific CSCs. A short summary of some prominent CSC markers showing prominent prognostic approaches towards specific CSC models is also pointed out (also in **Table 3**).

**Table 3:** A summarized form of certain CSC markers expressed on their target CSCs.

CSC marker	Distribution in CSCs	References
**Intracellular markers**		
ALDH1	AML, Bladder, Breast, Colorectal, Gastric, Glioma, HCC, Lung, Oesophageal squamous cell carcinoma, PDAC, Prostate, Renal cell carcinoma	(349–361)
NANOG	Breast, Colorectal, Gastric, Glioma, HCC, Head and Neck squamous cell carcinoma, Lung, Oral Squamous cell carcinoma, Ovarian	(362–372)
Oct4	Bladder, Breast, Glioma, HCC, Lung, Medulloblastoma, Melanoma, Oesophageal squamous cell carcinoma, Oral squamous cell carcinoma, Osteosarcoma, Ovarian, Pancreatic, Prostate	(373–377)
**Surface markers**		
EpCAM	Breast, Colorectal, Gastric, HCC, Lung, Pancreatic, Prostate	(325, 352, 378, 379)

## Conclusion

**Figure 3:**
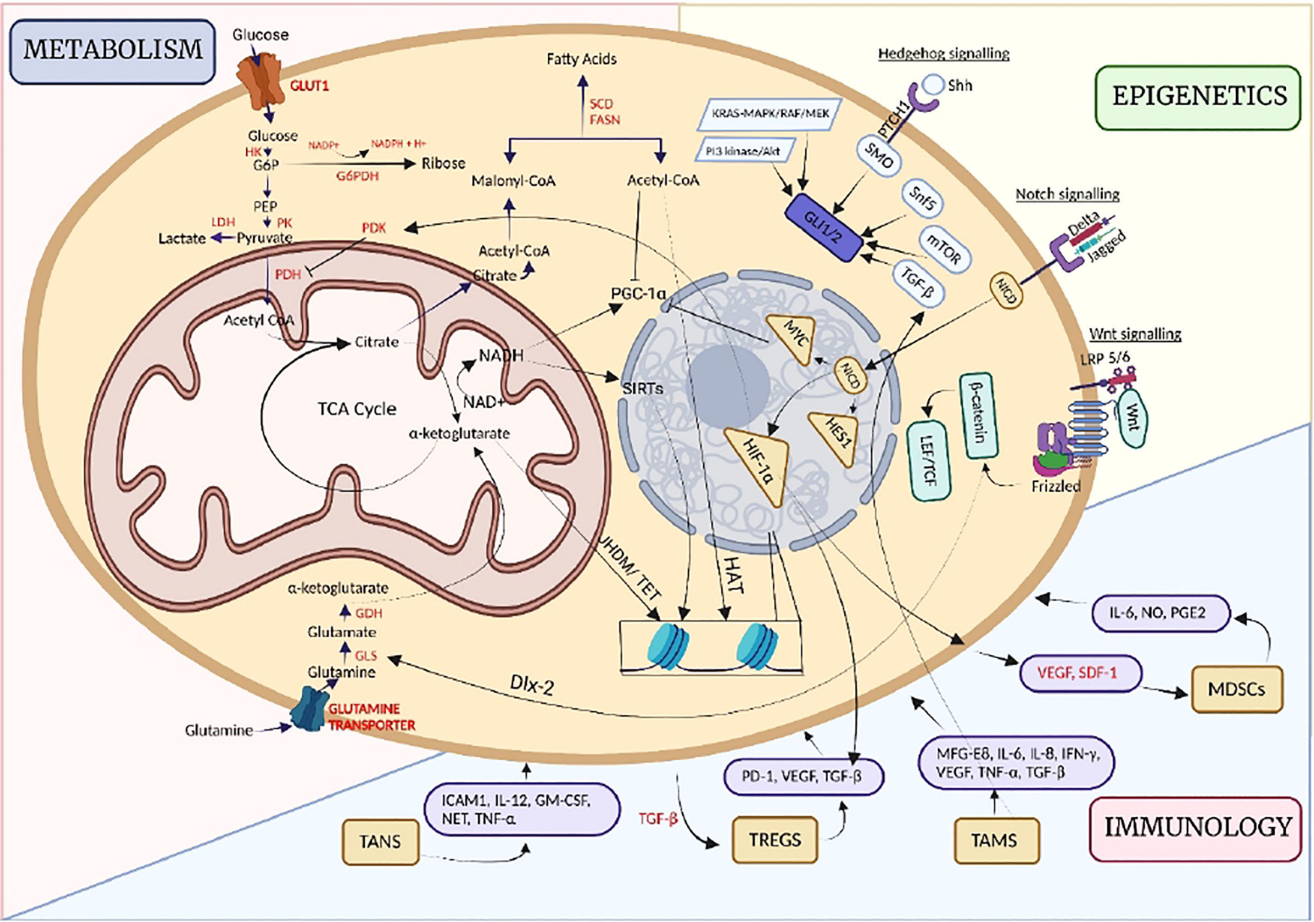
Pictorial representation of the different functional domains of Cancer Stem Cells. Epigenetic mechanisms have a profound influence on regulating the activities of key metabolic enzymes. Several of the enzymatic reactions, in turn, produce metabolites and onco-metabolites that in turn orchestrate key signaling pathways. TET ( Ten Eleven translocases) are one such mediator. Epigenetic mechanisms in turn also regulate mitogenic signals and alter the way cell interacts with their surroundings.

With advances in technology, our understanding of CSCs and cancer have strengthened dramatically. The crosstalk among metabolic, epigenetic, and immunological aspects involved in CSC generation and recurrence substantially enhance tumorigenesis and metastasis. Several shreds of evidence have indicated that it is not just a specific domain that orchestrates tumor formation; instead, each and every domain has its role in developing the CSC niche (**Figure 3**). Though the origin of CSC has always been a contentious matter, it can be postulated that CSCs show stemness features and are resistant to chemo/radiotherapy alone. Thus, targeting the CSC population would give better insights into anti-tumor treatment, thereby eradicating cancer. Identifying the CSC with specific cell surface markers as mentioned before, forms fundamentally the first step in targeting the CSC. There are several universal CSC markers, with a few been extensively used in multiple studies.

**Figure 4:**
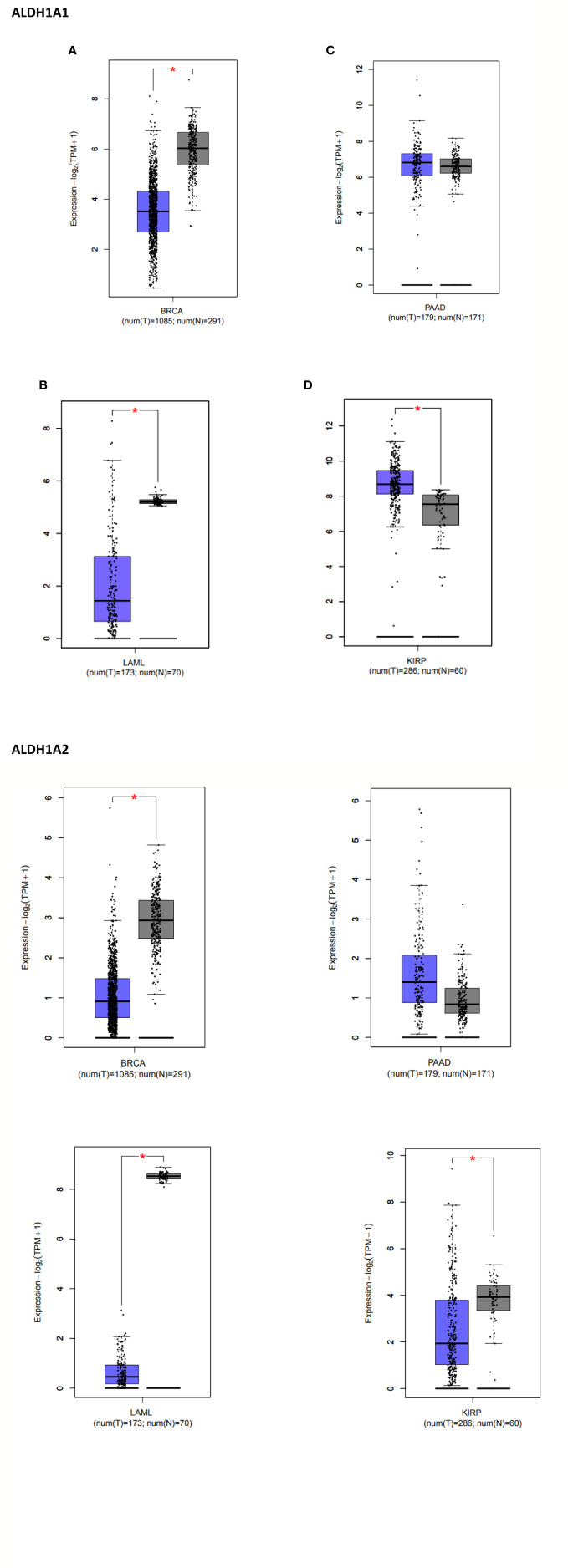
ALDH1A1 is differentially expressed in a cancer specific manner with few cancers showing downregulation(LAML, BRCA, **A, B**), upregulation (KIRP, **C**) and no changes (PAAD, **D**). TCGA data was analyzed using GEPIA tools.

ALDH1A1 and ALDH1A2 are used to isolate CSC populations, with their expression related to stemness. They seems to be associated with different cancers in a context specific manner (**Figure 4**). While it is downregulated in few cancers, it is upregulated in few others indicating a complex interplay between cells and environment.

A coordinated effort has been made to present all the interdisciplinary domains of CSCs under the purview of a single review in an elegant manner. The various dimensions of CSC, viz metabolic, epigenetic, and immunogenic interactions of the CSC population with the host organism, have been elucidated. Further discussion has also been made on treating these CSC subsets and cancer populations using novel therapeutic methods. We believe that the combined therapeutic trials’ success will definitely provide a promising path to pursue in future. However, the authors feel that there are still areas that require further investigation and research. In particular:

- To obtain a better understanding of the mechanisms involved in the driving of CSC initiation from normal naïve cells.
- To be able to differentiate the mechanisms followed by CSCs, naïve cancer cells, and normal stem cells.
- To ascertain the relative inter-contribution of the metabolic, epigenetic, and immunogenic domains, to CSC generation and determine if any particular domain can eradicate the transformed state of cancer.
- A combined approach to study the intra/inter CSC domains to target CSC populations.
- Delineating the different metaboepigenetic and other immunogenic approaches to circumvent the limitations caused due to less research in the field.

## Data availability statement

The raw data supporting the conclusions of this article will be made available by the authors, without undue reservation.

## Author contributions

OS drafted the manuscript and made the images with assistance from TS, JT, MAl, MAb and KP. MAl and MAb provided critical scientific and technological inputs and assistance. RD and SK conceptualized and oversaw the entire work. All authors contributed to the article and approved the submitted version.

## Funding

SK acknowledge Department of Health Research (DHR)–Grant in aid (2020-9644) for funding. TS acknowledge Council of Scientific and Industrial Research (CSIR) for fellowship (564/(CSIR-UGC NET JUNE 2019). JT acknowledge Indian Council of Medical Research (ICMR) for financial support (2021-11320/F1). MSA and MA extend their appreciation to the Deanship of Scientific Research at King Khalid University (KKU) for funding this work through the Research Group Program Under the Grant Number:(R.G.P.2/248/43).

## Conflict of interest

The authors declare that the research was conducted in the absence of any commercial or financial relationships that could be construed as a potential conflict of interest.

## Publisher’s Note

All claims expressed in this article are solely those of the authors and do not necessarily represent those of their affiliated organizations, or those of the publisher, the editors and the reviewers. Any product that may be evaluated in this article, or claim that may be made by its manufacturer, is not guaranteed or endorsed by the publisher.

## Abbreviations

CSC, Cancer Stem Cell; ALDH, Aldehyde dehydrogenase; AML, Acute myeloid leukemia; BLBC, Basal,like breast cancer; CRC, Colorectal Cancer; CXCL, Chemokine (C,X,C motif) ligand; DNMT, DNA methyltransferase; Dlx,2, Distal-less homobox-2; FAO, Fatty Acid Oxidation; GBM, Glioblastoma multiforme; HCC, Hepatocellular Carcinoma; HH, Hedgehog; HIF, Hypoxia-inducible factor; IFN-γ, Interferon-gamma; JHDM, Jumonji-C domain-containing Histone demethylase; mAB, Monoclonal antibody; MDSC, Myeloid,derived suppressor cells; MCT, Monocarboxylate transporters; mTORC, Mammalian Target of Rapamycin Complex; NET, Neutrophil Extracellular Trap; OXPHOS, Oxidative Phosphorylation; PDAC, Pancreatic Ductal Adenocarcinoma; PDK, Pyruvate Dehydrogenase kinase; PGC-1α, Peroxisome proliferator-activated receptor gamma co-activator 1α; PKM2 – Pyruvate kinase M2; PPP, Pentose Phosphate Pathway; ROS, Reactive Oxygen Species; TAM, Tumor,associated macrophages; TAN, Tumor,associated neutrophils; TCA, Tricarboxylic acid; TET, Ten,eleven translocation proteins; TGF β, Transforming growth factor β; TNBC, Triple,negative breast cancer; TNF α, Tumor necrosis factor α; Tregs, Regulatory T cells.
